# The Efficacy of Plant Extracts Against Key Food-Borne Pathogens: A Mechanistic, Applications, and Advances

**DOI:** 10.3390/microorganisms14030621

**Published:** 2026-03-10

**Authors:** Seham M. Al Raish

**Affiliations:** Department of Biology, College of Science, United Arab Emirates University, Al Ain 15551, United Arab Emirates; seham.alraish@uaeu.ac.ae

**Keywords:** plant extract, food pathogens, preservation, antimicrobial

## Abstract

The dual challenge of rising antimicrobial resistance and consumer demand for natural preservatives threatens global food safety. In this context, plant-derived polyphenolic extracts offer a potent and sustainable solution. These compounds exhibit potent activity against key food-borne pathogens responsible for diseases ranging from diarrhea to gastrointestinal ulcers, as well as pathogen-associated inflammatory conditions linked to long-term gastrointestinal complications. This review synthesizes current research on the efficacy of polyphenolic extracts as antimicrobial agents. I detail their diverse mechanisms of action, evaluate their potential for medical applications, and discuss their integration into advanced food preservation systems to meet modern safety and sustainability standards. Collectively, these insights highlight the potential of plant-derived polyphenolic extracts as sustainable tools for improving food safety in modern food systems.

## 1. Introduction

Food-borne pathogens remain a significant global public health concern, contributing to substantial illness, mortality, and economic loss. According to the World Health Organization (WHO), approximately 600 million people experienced foodborne infections in 2010, resulting in more than 420,000 deaths and an estimated 550 disability-adjusted life years (DALYs) per 100,000 population [1,2]. Importantly, this burden is not limited to low-income regions; higher-income countries, including those in Europe, report 41–49 DALYs per 100,000 population attributable to foodborne diseases [3]. The actual burden of these pathogens is often underestimated due to underreporting and insufficient documentation [4]. Clinical manifestations range from vomiting and diarrhea to severe neurological complications and, in extreme cases, death [5]. Beyond individual health, foodborne infections negatively impact productivity and food system stability and impose significant economic costs [6].

In the Middle East, gastrointestinal infections are often linked to pathogens such as *Salmonella Typhi*, *Shigella*, *Campylobacter jejuni*, *Escherichia coli*, rotavirus, hepatitis A virus, and emerging or regionally relevant microorganisms, including *Aeromonas*, *Yersinia enterocolitica*, and *Brucella* spp. [7]. Many of these pathogens produce metabolites that influence chronic disease risk. For example, toxins from *Salmonella* spp., *Campylobacter* spp., *Listeria monocytogenes*, and *Escherichia coli* can be associated with foodborne exposure and activate inflammatory and oncogenic signaling pathways, including Nuclear factor kappa B (NF-κB), interfere with tumor suppressor genes, and contribute to inflammation-driven carcinogenesis [8,9,10,11].

Environmental and technological changes further intensify foodborne disease risks. Climate change alters pathogen survival, distribution, and transmission dynamics by modifying temperature, humidity, and host–pathogen interactions [12,13]. Shifts in food production and processing systems have also enabled the emergence of new pathogens and reshaped the epidemiology of traditional foodborne illnesses [4]. Meanwhile, antimicrobial resistance (AMR), accelerated by widespread antibiotic misuse in medical practice and food production, threatens the efficacy of standard treatments and increases the likelihood of severe and persistent infections [14,15,16]. These challenges highlight the need for safer, sustainable, and effective alternatives to conventional chemical preservatives.

Medicinal plants have gained renewed scientific attention as promising sources of antimicrobial agents. Their bioactive constituents, particularly polyphenols, flavonoids, tannins, and essential oils, exhibit diverse biological functions, including antibacterial, antioxidant, and anti-inflammatory activities [17,18,19,20]. Notably, many plant-derived compounds act synergistically, enabling them to overcome bacterial resistance mechanisms that compromise the effectiveness of conventional antibiotics. While numerous plants have been studied extensively, others remain remarkably underexplored. For example, *Ruta graveolens* contains more than 230 identified phytochemicals, including coumarins, quinolone alkaloids, acridone alkaloids, flavonoids, and volatile oils. These compounds exhibit potent antibacterial, antifungal, anti-*Helicobacter pylori*, anti-inflammatory, and antiproliferative activities, underscoring the vast untapped potential of lesser-known botanical species [21].

Although several reviews have explored plant-derived antimicrobials, most remain limited in scope and do not provide a broad comparative synthesis [22,23,24,25,26,27,28,29]. For example, Chan et al. (2018) [20] evaluated polyphenols from selected spices but did not assess their relevance across diverse food-borne pathogens. Gengatharan and Rahim (2023) [30] focused specifically on the application of clove extracts in packaging systems, while Idowu et al. (2021) [31] discussed clove-derived compounds mainly within dairy applications. These studies demonstrate that the existing literature often focuses on single plants or narrow applications and fails to integrate mechanisms, therapeutic relevance, and food-preservation potential across multiple medicinal species. This review addresses that gap by presenting an integrated overview of five key plant species: clove, pomegranate, cranberry, garlic, and cinnamon, together with their principal bioactive compounds, mechanisms of antibacterial action, and emerging advances such as nano-enabled delivery systems.

Although several reviews have addressed individual plant extracts or specific applications, the existing literature remains fragmented and largely plant-specific. In contrast, this review provides an integrated synthesis of five widely studied medicinal plants, comparing their bioactive compounds, shared and distinct antimicrobial mechanisms, translational relevance to food systems, and emerging advances such as nano-enabled delivery strategies.

## 2. Methodology

This manuscript is a structured narrative review that synthesizes current knowledge on plant-derived extracts relevant to food-borne pathogens and food safety applications. A comprehensive literature search was conducted across PubMed, Scopus, Web of Science, and Google Scholar.

Search terms included combinations of the following keywords: “plant extracts,” “polyphenols,” “antimicrobial activity,” “food-borne pathogens,” “food preservation,” “minimum inhibitory concentration (MIC),” “antioxidant activity,” and “nanoencapsulation.”

Peer-reviewed articles published primarily within the last 15 years were considered to ensure up-to-date coverage. Earlier foundational studies were included where necessary to provide a mechanistic context. Studies were selected based on relevance to antimicrobial mechanisms, food-system applicability, pathogen control, antioxidant properties, and emerging preservation technologies.

Non-peer-reviewed sources, studies lacking experimental validation, and publications not directly related to food-borne pathogens or food applications were excluded.

As a narrative review, this work does not follow a formal systematic review protocol; however, a structured and criteria-based selection strategy was employed to enhance transparency and minimize selection bias.

## 3. Bioactive Compounds and Extraction Characteristics

This section summarizes the principal bioactive compounds of five selected medicinal plants and highlights how extraction methods, solvent polarity, and processing conditions influence compound recovery and stability. Emphasis is placed on compositional characteristics and extraction efficiency, while antimicrobial mechanisms are discussed separately in Section 4.

### 3.1. Clove (Syzygium aromaticum)

Clove is characterized by a high content of volatile phenolic compounds, primarily eugenol, eugenol acetate, β-caryophyllene, and α-humulene [30,32,33,34]. These compounds are commonly extracted through hydrodistillation, Soxhlet extraction, ethanol or methanol maceration, and ultrasound-assisted techniques. Extraction efficiency depends strongly on solvent polarity, temperature, and plant matrix characteristics. High-polarity solvents such as ethanol and methanol have been shown to yield significantly higher total phenolic content and greater eugenol recovery compared to non-polar solvents [35]. Reported extraction yields range between 8% and 18%, depending on extraction parameters [35]. Because eugenol is moderately polar, solvent selection directly influences both yield and bioactive concentration, which in turn affects downstream functional applications [35].

### 3.2. Pomegranate (Punica granatum L.)

Pomegranate peel contains abundant ellagitannins, including punicalagin, ellagic acid, anthocyanins, and flavonoids [36,37,38,39]. Extraction is typically performed using aqueous, ethanolic, or methanolic solvents. Ethanol-based extraction systems demonstrate higher recovery of punicalagin and ellagic acid due to favorable solubility profiles [37,38]. Extraction yields from dried peel can reach 30–40%, depending on solvent ratio, drying conditions, and extraction temperature [37]. Thermal stability studies indicate that phenolic-rich extracts retain substantial activity after heat exposure, supporting their suitability for food-processing environments [37].

### 3.3. Cranberry (Vaccinium macrocarpon) Pomace Extract

Cranberry pomace is rich in proanthocyanidins (PACs), anthocyanins, and phenolic acids [40,41,42,43,44]. PAC extraction is optimized using acidified ethanol or aqueous ethanol systems, which enhance recovery of oligomeric proanthocyanidins [40]. The concentration and structural profile of PACs depend on fruit maturity, cultivar, and postharvest handling [44]. These phenolic fractions demonstrate strong oxidative stability and maintain functional properties under controlled storage conditions [40].

### 3.4. Garlic (Allium sativum)

Garlic contains sulfur-containing bioactive compounds, including allicin, diallyl sulfides, S-allyl cysteine, and related organosulfur derivatives [45,46]. Allicin is produced enzymatically from alliin upon tissue disruption through alliinase activation [47]. Extraction methods include aqueous extraction, ethanol extraction, mechanical pressing, and steam distillation for oil isolation. Steam-distilled garlic oil contains a broader range of lipid-soluble organosulfur compounds compared to aqueous extracts [48]. Processing conditions, such as aging (black garlic production), significantly alter sulfur compound profiles and increase phenolic and antioxidant content [42,49].

### 3.5. Cinnamon (Cinnamomum zeylanicum)

Cinnamon bark contains cinnamaldehyde, catechins, procyanidins, and other flavan-3-ol derivatives [50,51,52]. Essential oils are typically obtained by steam distillation, whereas solvent extraction yields phenolic-rich fractions. Extraction efficiency depends on bark maturity, solvent polarity, and distillation conditions [50]. Stability assessments indicate that cinnamon-derived phenolics retain functional properties under refrigerated storage and moderate thermal processing [53].

In the context of food safety, cinnamon-derived polyphenols are of interest for their reported antimicrobial activity against foodborne pathogens and their potential influence on intestinal barrier integrity following pathogen exposure. These properties may contribute to limiting pathogen colonization and supporting gastrointestinal resilience in food-related contexts (Table 1). Laboratory studies have reported inhibitory activity against spoilage molds under controlled experimental conditions (Table 2) [40].

## 4. Antibacterial Potential of Herbal Extracts

### 4.1. Clove (Syzygium aromaticum)

**Figure 1:**
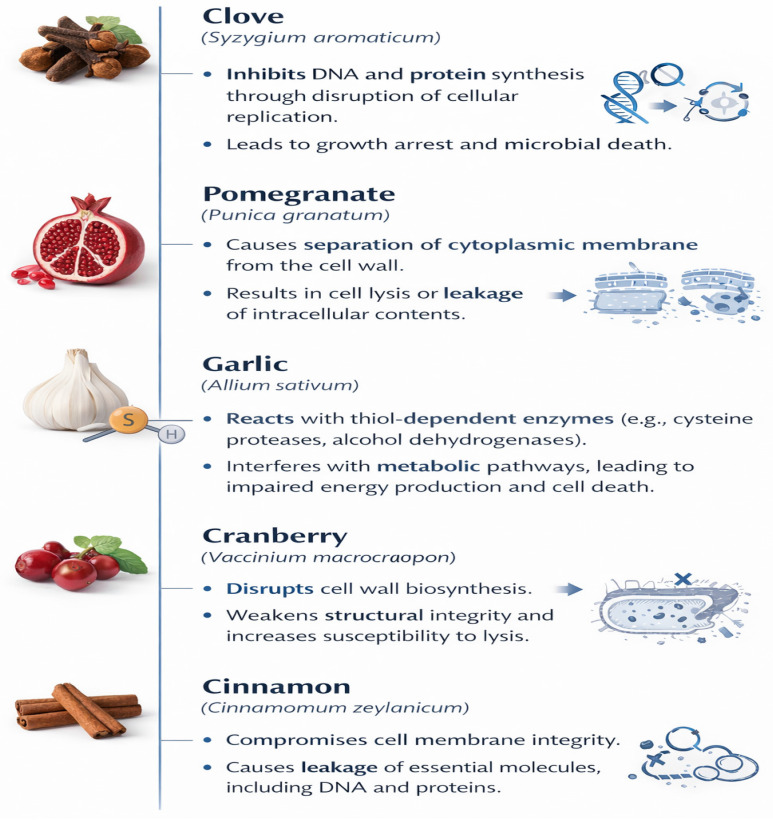
Antibacterial mechanisms of selected medicinal plant extracts. Mechanistic actions of key bioactive compounds from clove (*Syzygium aromaticum*), pomegranate (*Punica granatum*), garlic (*Allium sativum*), cranberry (*Vaccinium macrocarpon*), and cinnamon (*Cinnamomum zeylanicum*) against food-borne pathogens. Eugenol (clove) has been reported to disrupt cytoplasmic membrane structure and increase permeability in Gram-negative and Gram-positive bacteria, including *Escherichia coli* and *Staphylococcus aureus*. Punicalagin and ellagitannins (pomegranate) have been associated with membrane destabilization and interference with enzymatic function in pathogens such as *Salmonella Typhi* and *Listeria monocytogenes*. Allicin (garlic) has been shown to react with thiol-containing enzymes, potentially impairing key metabolic pathways in *Helicobacter pylori* and other bacteria. Proanthocyanidins (cranberry) are suggested to interfere with adhesion and biofilm formation in *Escherichia coli* and *Streptococcus mutans* via anti-adhesion and quorum-sensing modulation. Cinnamaldehyde (cinnamon) has been reported to affect membrane integrity and may interfere with ATP synthesis and virulence-associated pathways in bacteria, including *L. monocytogenes*. Together, these mechanisms illustrate the multi-target nature of plant-derived antimicrobial compounds (Table 2).

The antibacterial activity of clove is primarily attributed to eugenol and related phenolic compounds [66]. Clove essential oil has demonstrated inhibitory effects against *Staphylococcus aureus*, *Escherichia coli*, *Yersinia enterocolitica*, and *Bacillus cereus* in model food systems (Table 2) [30,40]. Additional activity has been reported for eugenol acetate and sesquiterpenes such as α-humulene and β-caryophyllene [45,64,67]. The underlying antimicrobial mechanisms are summarized in Section 4.6 and illustrated in Figure 1. Practical efficacy depends on food matrix composition and processing parameters [30,40].

### 4.2. Pomegranate (Punica granatum)

Pomegranate peel extracts exhibit antibacterial activity mainly due to ellagitannins, punicalagin, and ellagic acid [47]. These extracts have demonstrated inhibitory effects against major food-borne pathogens, including *Salmonella Typhi*, *Listeria monocytogenes*, *Staphylococcus aureus*, and *Escherichia coli* [35,50,51,52,53,58]. In food-related applications, pomegranate peel extract has been shown to reduce microbial contamination in meat systems [51]. The principal antimicrobial mechanisms are summarized in Section 4.6. Their effectiveness in food systems is influenced by concentration, matrix composition, and sensory constraints [47,51].

### 4.3. Cranberry (Vaccinium macrocarpon)

Cranberry-derived proanthocyanidins (PACs) exert antimicrobial activity primarily through anti-adhesion and anti-biofilm mechanisms rather than direct bactericidal effects [37]. Cranberry extracts have demonstrated inhibitory effects against antibiotic-resistant *Escherichia coli*, including CTX-M-15 ESBL-producing and multidrug-resistant strains [39,59,68], as well as Staphylococcus aureus in food systems [38]. These anti-virulence and biofilm-modulating properties are associated with interference in bacterial adhesion and quorum-sensing pathways, as consolidated in Section 4.6 and Figure 1. Such properties highlight their relevance in controlling pathogen persistence in food environments [38,39,59,68].

### 4.4. Garlic (Allium sativum)

Garlic exhibits broad-spectrum antibacterial activity due to allicin and other organosulfur compounds [60,61,62]. Garlic oil has demonstrated antimicrobial effects against *Helicobacter pylori* and various Gram-positive and Gram-negative food-borne pathogens [44,69]. The activity of garlic-derived compounds has been associated with interference in thiol-dependent enzyme systems and metabolic pathways [70], as summarized in Section 4.6. Antimicrobial performance varies depending on formulation, extraction method, and processing conditions [44,62].

### 4.5. Cinnamon Oil (Cinnamomum zeylanicum)

Cinnamon’s antibacterial activity is mainly attributed to cinnamaldehyde and related phenolic compounds [48,67,71]. Cinnamon essential oil has demonstrated inhibitory effects against *Listeria monocytogenes*, *Escherichia coli*, and mycotoxin-producing fungi such as *Aspergillus flavus* and *A. parasiticus* [72,73]. In meat systems, cinnamon demonstrated effective reduction in L. monocytogenes under controlled storage conditions (Table 2) [72]. These antimicrobial effects are associated with membrane-associated and metabolic interference mechanisms, as consolidated in Section 4.6 and Figure 1. Effectiveness is influenced by environmental and matrix factors [72,73] (Figure 1).

**Table 2:** Extraction methods and antimicrobial efficacy of selected plant extracts.

Plant	Extraction Method	Food-Relevant Antimicrobial Findings	Ref.
Clove	Hydrodistillation, ethanol extraction	MIC: 0.21–1.67 μL/mL; reduced bacterial counts in model food systems	[30,40]
Pomegranate	Ethanolic peel extraction	Reduced *Salmonella* contamination; effective meat decontamination	[50,51]
Cranberry	Acidified ethanol extraction	Inhibited ESBL-*E. coli* virulence; reduced *S. aureus* in cooked meat	[39,51,52]
Garlic	Steam distillation (oil)	Garlic oil more effective than powder against *H. pylori*	[62]
Cinnamon	Steam distillation	Reduced *L. monocytogenes* by ~2 log CFU/g in meat systems	[64]

### 4.6. Practical Relevance and Integrated Antimicrobial Mechanisms

Although MIC and MLC values indicate antimicrobial potency under laboratory conditions, their direct translation to food systems remains limited. The efficacy of plant extracts in real foods is strongly influenced by food matrix interactions, in which proteins, lipids, and carbohydrates can bind or neutralize bioactive compounds, thereby reducing antimicrobial activity [19,33,74]. Sensory constraints also limit the practical concentrations of extracts, as higher levels of clove, garlic, cinnamon, and pomegranate may introduce intense flavors, aromas, or color changes that are unacceptable in consumer products [31,66]. In addition, processing conditions, including pH, water activity, heat treatment, and storage parameters, can substantially affect the stability and effectiveness of plant extracts in situ [33,64,75]. Therefore, MIC and MLC values should be interpreted within the context of realistic food applications, where antimicrobial performance depends on microbial sensitivity as well as the physicochemical properties and sensory limitations of the food matrix.

Across the five medicinal plants reviewed, several shared antimicrobial patterns emerge. Many phenolic compounds have been reported to affect bacterial membrane integrity and permeability, while others are associated with interference in enzyme systems and metabolic pathways. Rather than acting through a single target, these bioactives appear to exert multi-target effects that collectively impair bacterial survival and virulence [54,65,73,76]. Many compounds, including allicin, eugenol derivatives, and proanthocyanidins, also interfere with bacterial enzyme systems, particularly those involving thiol-dependent metabolic pathways, thereby suppressing energy production and essential cellular functions [45,46,58].

Additionally, cranberry PACs, pomegranate tannins, and cinnamon phenolics demonstrate anti-biofilm activity, inhibiting adhesion, extracellular polymeric substance (EPS) formation, and biofilm maturation in both Gram-positive and Gram-negative bacteria [40,41,76]. Several phytochemicals further disrupt quorum-sensing signaling, reducing virulence gene expression and impairing bacterial communication necessary for coordinated pathogenic behavior [20,42].

Together, these overlapping mechanisms highlight the integrative and multi-targeted nature of plant-derived compounds, providing a mechanistic rationale for their potential use in food preservation and antimicrobial interventions. To minimize redundancy, detailed plant-specific mechanisms are not restated in each subsection; shared antimicrobial pathways are consolidated here.

## 5. Antioxidant Potential of Herbal Extract

Oxidative degradation contributes to reduced shelf life and quality in food systems. Plant-derived phenolic compounds have therefore been investigated as natural alternatives to synthetic antioxidants such as butylhydroxyanisole (BHA) and butylhydroxytoluene (BHT) [63]. The antioxidant efficacy of selected plant extracts is supported by quantitative experimental data.

Clove extract exhibits strong antioxidant activity, achieving 95.2% DPPH radical scavenging at 0.5 mg/mL and 72.3% metal-chelating activity at 1 mg/mL, exceeding the performance of BHA and BHT under comparable conditions [63].

Pomegranate peel extract demonstrates 70–83% DPPH inhibition at concentrations of 100–200 µg/mL, with total phenolic content ranging between 249 and 320 mg GAE/g. Approximately 66% of antioxidant activity is retained after exposure to 180 °C for 80 min, indicating strong thermal stability during food processing [47,77].

Cranberry pomace extracts reduce reactive oxygen species (ROS) by 42% and increase superoxide dismutase (SOD) activity by 35% in cellular models challenged with Salmonella Enteritidis, demonstrating cytoprotective antioxidant effects [78].

Garlic extracts reduce malondialdehyde (MDA) levels by 54–61% and increase SOD and catalase (CAT) activity by 1.7–2.3-fold in vivo, reflecting significant oxidative stress reduction [55].

Cinnamon extracts demonstrate DPPH scavenging values of 82–89% and ferric reducing antioxidant power (FRAP) values between 410 and 525 μmol Fe^2+^/g extract [40], supporting their free-radical neutralizing capacity in food matrices.

## 6. Applications

Food-borne pathogens primarily exert their health impacts through gastrointestinal infections, inflammation, and disruption of intestinal barrier integrity following consumption of contaminated food. The following subsections, therefore, discuss gastrointestinal outcomes in the context of food-borne microbial exposure and host–pathogen interactions, rather than as general therapeutic or clinical treatment claims. This focus maintains alignment with the food safety scope of the present review.

### 6.1. Gastrointestinal Infectious Diarrheal Disorders

Studies validated the traditional use of medicinal plants for treating diarrhea by investigating the biological activity of their extracts. These extracts have antispasmodic effects, delay intestinal transit, suppress gut motility, stimulate water adsorption, and reduce electrolyte secretion, primarily through tannins and flavonoids [77]. Diarrhea is a common illness associated with abnormalities in gut immune activity and homeostasis [79]. A study revealed that the aqueous extract of pomegranate peels contains substances that reduce diarrhea by inhibiting intestinal motility and fluid accumulation [80]. Cinnamon is a phytomedicinal plant with antibacterial and anti-inflammatory properties that can help control complications of *H. pylori* treatment and improve the efficacy of antibiotics [81]. A study found that cinnamon water extract contains coumarin and cinnamic acid, which are responsible for relieving diarrhea symptoms by altering the intestinal environment [79].

### 6.2. Gastrointestinal Infectious Ulcer Disorders

Gastric ulcer is a common disease that may develop following infection, inflammation, or pathogen-associated gastrointestinal stress from contaminated food [82]. Clovinol in clove demonstrated antioxidant and anti-inflammatory properties, inhibiting carrageenan-induced paw swelling in mice. It also showed anti-ulcerogenic activity, upregulated antioxidant levels, and reduced lipid peroxidation in ulcer-induced rats, suggesting protective effects on gastric mucosal integrity [83]. Pomegranate has demonstrated its ability to protect against and modulate gastric ulcer development. Oral administration of cinnamon oil showed a gastroprotective effect against gastric ulcer, with a significant increase in gastric levels of enzymatic and non-enzymatic antioxidants, namely CAT, SOD, GSH-Px, and GSH, and a combined reduction in malondialdehyde (MDA) levels [84]. Cinnamon’s gastroprotection has been associated with inhibition of basal gastric secretion, stimulation of mucus secretion, and increased nonprotein-sulfhydryl concentration, likely due to its antioxidant properties [63]. A study on indomethacin-induced gastric ulcers in rats found that the anti-inflammatory and antioxidant properties of cinnamon extracts protected the gastric mucosa and improved histological outcomes [85].

In the context of food safety, gastric mucosal damage and inflammation may occur following infection with food-borne pathogens or ingestion of contaminated food. Several plant-derived polyphenols discussed in this review have been shown to modulate oxidative stress, inflammatory signaling, and epithelial integrity within the gastrointestinal tract. These effects are particularly relevant in food-borne infections, where pathogen-induced inflammation and mucosal damage contribute to disease severity and prolonged recovery. Therefore, the biological activities of plant extracts are discussed here in relation to gastrointestinal infection and host–pathogen interactions, rather than as generalized clinical treatment claims.

### 6.3. Food Preservation

Clove extracts are widely applied for pharmacological benefits, food flavoring, and preservation [86], as well as in food packaging, in dairy products, and for preservation of processed food, meat, poultry, seafood products, and vegetables [87]. Clove’s essential oils were used to develop active packaging films with strong antibacterial activity against *Staphylococcus aureus* and *Escherichia coli* for food preservation in dairy products [31].

A recent study added a high-ethanolic extract of pomegranate peels to functional yogurt and compared it to control yogurt under normal environmental conditions. The fortified yogurt showed the best results in pH, titratable acidity, reduced syneresis, increased water-holding capacity, improved color parameters, and increased survival of *Lactobacillus bulgaricus* and *Streptococcus thermophilus* [66]. Another study developed ginger, turmeric, and pomegranate peel extracts, which were incorporated into pasteurized milk; this study found that all extracts exhibited inhibition zones against both pathogenic Gram-negative and Gram-positive bacteria and pathogenic fungi. However, pomegranate peel extract has the highest inhibitory activity among plant extracts [74]. Cranberry extract was used as an antibiofilm additive in chitosan-based films intended for food packaging applications, with antioxidant properties [88].

One of the most significant approaches in food preservation strategies is the use of nanomaterials, which offer many benefits across fields such as food packaging and materials processing to improve sustainability [89]. Encapsulating plant extracts into nanomaterials has attracted extensive research attention across various fields, including the food and agriculture sectors, as they are more stable and efficient and offer advanced functionalities [90]. Incorporation of clove essential oil nanoemulsions into a pullulan-sodium alginate composite film via ultrasound is highly effective for preserving cherries and mushrooms [64]. A recent study used nanocapsules to encapsulate pomegranate peel extract in meat, ensuring gradual release of bioactive compounds and sustained inhibition of lipid oxidation and microbial proliferation, thereby improving shelf life compared to free extract treatment [91].

Although nanoencapsulation and nanoemulsion technologies offer improved stability, enhanced bioavailability, and controlled release of plant extracts, several challenges limit their widespread translation into food systems. Regulatory acceptance remains uncertain, as nano-enabled formulations often fall outside established food-grade classifications and require extensive evaluation before approval for commercial use [89]. Long-term safety data are limited, particularly regarding nanoparticle accumulation, interactions with the gastrointestinal environment, and potential impacts on gut microbiota composition [90]. Additionally, the scalability of nano-formulation technologies, including high-pressure homogenization, ultrasonication, and microencapsulation, is constrained by high production costs, energy requirements, and the need for specialized equipment that is not widely available in food-processing facilities [64,89]. Moreover, the performance of nanoencapsulated extracts may vary under real processing conditions, as pH, temperature, and storage stability can significantly affect particle integrity and the release of bioactive compounds [64,91]. Addressing these regulatory, safety, and manufacturing limitations will be essential for enabling the practical adoption of nano-enabled plant extracts in food preservation.

### 6.4. Safety and Regulatory Considerations

The incorporation of plant-derived extracts into food systems also requires consideration of safety and regulatory frameworks. Many essential oils and phenolic-rich extracts, such as clove, pomegranate, garlic, cranberry, and cinnamon, are classified as Generally Recognized as Safe (GRAS) when used within established limits; however, their safety can vary with concentration, formulation, and intended food application [66,86]. Toxicological studies indicate that high doses or prolonged exposure may affect cellular redox balance, gastrointestinal function, or metabolic pathways, highlighting the importance of dose optimization and product-specific evaluation [84,92].

For nano-formulated plant extracts, regulatory oversight is more complex. Current guidelines from food safety authorities require a detailed assessment of nanoparticle characteristics, including size distribution, migration potential, digestion behavior, and long-term biological accumulation [89,90]. Challenges remain due to the absence of universally accepted regulatory criteria, limited long-term toxicological data, and uncertainties regarding consumer acceptance. Furthermore, the classification of nanoencapsulated bioactives within existing food-additive regulations is still evolving, creating barriers for commercial adoption.

Together, these considerations emphasize that both conventional and nano-enabled plant extract applications must balance biological efficacy with compliance, safety validation, and regulatory clarity before implementation in food systems.

## 7. Conclusions

Food-borne pathogens are microorganisms that pose significant public health risks and food safety hazards through contamination. Plant extracts play a multifunctional role against foodborne pathogens through their antimicrobial and antioxidant activities. This review summarizes antimicrobial mechanisms and their relevance to controlling food-borne pathogens, limiting gastrointestinal infection, and supporting food safety applications. Plant extracts show promising applications in food preservation. Recently, advances in nanotechnology, such as encapsulation and nanoemulsions, have provided crucial solutions to overcome inherent challenges, including volatility and interactions with the food matrix. Further research is needed to expand the utility of plant extracts for controlling food spoilage and associated pathogens. The novelty of this review lies in its integrative framework, which bridges mechanistic antimicrobial evidence with realistic food-system applicability and emerging preservation technologies, rather than reiterating isolated antimicrobial observations.

## 8. Challenges and Future Perspectives in the Application of Plant Extracts in the Food Industry

Despite the promising antimicrobial and antioxidant properties of plant-derived extracts, several challenges limit their widespread adoption in food systems. One major issue is the lack of standardization in extract preparation, as variability in plant species, extraction methods, solvent systems, and environmental growing conditions can lead to significant differences in phytochemical composition and bioactivity. Establishing standardized protocols and identifying marker compounds for quality control will be crucial for ensuring consistency and efficacy in commercial applications.

Regulatory hurdles also pose a barrier. Approval processes for natural antimicrobials vary across countries, and plant extracts must demonstrate safety, stability, and reproducibility before they are incorporated into food products. Clear regulatory frameworks and harmonized international standards are needed to facilitate the integration of plant-based preservatives into global food markets.

Another important consideration is potential toxicity. While many plant extracts are generally recognized as safe, high concentrations or prolonged exposure may pose toxicological risks or interact with other food components. Comprehensive toxicological assessments, including in vivo studies and long-term exposure evaluations, are essential to establish safe usage levels.

Additionally, the incorporation of plant extracts can influence sensory attributes such as flavor, aroma, and color, which may affect consumer acceptance. Research into encapsulation technologies, controlled-release systems, and formulation strategies may help mitigate undesirable sensory effects while preserving antimicrobial functionality.

A comparative evaluation of the five reviewed plant extracts highlights important differences in antimicrobial behavior and practical suitability across food systems. Clove and cinnamon essential oils demonstrate relatively strong, rapid bactericidal activity in meat and poultry matrices, supported by low reported MIC values and effective reduction in *Listeria* *monocytogenes* and *Escherichia coli* in model systems [30,40,72]. However, their high volatility and intense aroma may limit application levels due to sensory constraints [31,66]. Pomegranate peel extract offers broader compatibility in dairy and fermented products, demonstrating effective pathogen inhibition while contributing antioxidant stability and improved physicochemical properties in yogurt and milk systems [50,66,74]. Cranberry extracts, particularly proanthocyanidin-rich fractions, exhibit comparatively stronger anti-adhesion and anti-biofilm properties rather than direct bactericidal effects, making them particularly suitable for packaging materials and surface decontamination strategies [39,51,52,59,68,88]. Garlic oil demonstrates potent antimicrobial activity against *Helicobacter pylori* and multiple Gram-positive and Gram-negative food-borne pathogens [44,62,69], yet formulation challenges related to odor intensity and compound instability may restrict its use in certain food matrices. These distinctions emphasize that plant extracts are not universally interchangeable; rather, their effectiveness depends on pathogen type, food composition, processing conditions, and sensory acceptability.

Looking ahead, advances in nanotechnology, encapsulation systems, and synergistic combinations of plant extracts may enhance stability, bioavailability, and targeted delivery in food matrices. Continued interdisciplinary research will be essential to overcome current challenges and realize the full potential of plant-derived compounds as sustainable, natural alternatives to synthetic preservatives.

## Figures and Tables

**Table 1:** Major bioactive compounds and target food-borne pathogens.

Plant	Main Bioactive Compounds	Target Food-Borne Pathogens	Ref.
Clove (*Syzygium aromaticum*)	Eugenol, eugenol acetate, α-humulene, β-caryophyllene	*S. aureus*, *E. coli*, *Y. enterocolitica*, *B. cereus*, molds	[40,41,42,54,55]
Pomegranate (*Punica granatum*)	Punicalagin, ellagic acid, tannins	*S. Typhi*, *L. monocytogenes*, *E. coli*, *S. aureus*, *K. pneumoniae*	[50,51,52,53,56,57]
Cranberry (*Vaccinium macrocarpon*)	Proanthocyanidins (PACs), anthocyanins	ESBL-*E. coli*, MDR-*E. coli*, *S. mutans*, *S. aureus*	[39,51,52,58,59]
Garlic (*Allium sativum*)	Allicin, organosulfur compounds	*H. pylori*, Gram-positive and Gram-negative foodborne pathogens	[60,61,62,63]
Cinnamon (*Cinnamomum zeylanicum*)	Cinnamaldehyde, catechins, procyanidins	*L. monocytogenes*, *E. coli*, *A. flavus*, *A. parasiticus*	[64,65]

## Data Availability

No new data were created or analyzed in this study. Data sharing is not applicable to this article.

## References

[1] Mortazavi N., Aliakbarlu J., Imani M. (2025). The Antibacterial Effects of Engineered Salmonella Phage PVP-SE1 Endolysin on the Planktonic Cells and Biofilms of Food-Borne Pathogens and Its Antibacterial Activity in Milk. Food Biosci..

[2] Pires S.M., Devleesschauwer B. (2021). Estimates of Global Disease Burden Associated with Foodborne Pathogens. Foodborne Infections and Intoxications.

[3] Lake I.R., Barker G.C. (2018). Climate Change, Foodborne Pathogens and Illness in Higher-Income Countries. Curr. Environ. Health Rep..

[4] Hamaideh S., Olaimat A., Al-Holy M., Ababneh A., Shahbaz H., Abughoush M., Al-Nabulsi A., Osaili T., Ayyash M., Holley R. (2024). The Influence of Technological Shifts in the Food Chain on the Emergence of Foodborne Pathogens: An Overview. Appl. Microbiol..

[5] Al-Seghayer M.S., Al-Sarraj F.M. (2021). The Outbreak of Foodborne Disease by Pathogenic Enterobacteriaceae Antimicrobial Resistance—A Review. Asian Food Sci. J..

[6] Adley C.C., Ryan M.P. (2025). The Nature and Extent of Foodborne Disease. Antimicrobial Food Packaging.

[7] Todd E.C.D., Murad S., Baydoun E., Daghir N. (2017). Foodborne Disease in the Middle East. Water, Energy & Food Sustainability in the Middle East.

[8] Mafe A.N., Büsselberg D. (2024). Impact of Metabolites from Foodborne Pathogens on Cancer. Foods.

[9] Jha N.K., Arfin S., Jha S.K., Kar R., Dey A., Gundamaraju R., Ashraf G.M., Gupta P.K., Dhanasekaran S., Abomughaid M.M. (2022). Re-Establishing the Comprehension of Phytomedicine and Nanomedicine in Inflammation-Mediated Cancer Signaling. Semin. Cancer Biol..

[10] Rahman M.d.M., Islam M.d.R., Shohag S., Ahasan M.d.T., Sarkar N., Khan H., Hasan A.M., Cavalu S., Rauf A. (2022). Microbiome in Cancer: Role in Carcinogenesis and Impact in Therapeutic Strategies. Biomed. Pharmacother..

[11] Zhang Y., Chen R., Zhang D., Qi S., Liu Y. (2023). Metabolite Interactions between Host and Microbiota during Health and Disease: Which Feeds the Other?. Biomed. Pharmacother..

[12] Awad D.A., Masoud H.A., Hamad A. (2024). Climate Changes and Food-Borne Pathogens: The Impact on Human Health and Mitigation Strategy. Clim. Change.

[13] Singh B.K., Delgado-Baquerizo M., Egidi E., Guirado E., Leach J.E., Liu H., Trivedi P. (2023). Climate Change Impacts on Plant Pathogens, Food Security and Paths Forward. Nat. Rev. Microbiol..

[14] Abukhattab S., Taweel H., Awad A., Crump L., Vonaesch P., Zinsstag J., Hattendorf J., Abu-Rmeileh N.M.E. (2022). Systematic Review and Meta-Analysis of Integrated Studies on Salmonella and Campylobacter Prevalence, Serovar, and Phenotyping and Genetic of Antimicrobial Resistance in the Middle East—A One Health Perspective. Antibiotics.

[15] Alsayeqh A.F., Baz A.H.A., Darwish W.S. (2021). Antimicrobial-Resistant Foodborne Pathogens in the Middle East: A Systematic Review. Environ. Sci. Pollut. Res..

[16] Escher N.A., Muhummed A.M., Hattendorf J., Vonaesch P., Zinsstag J. (2021). Systematic Review and Meta-analysis of Integrated Studies on Antimicrobial Resistance Genes in Africa—A One Health Perspective. Trop. Med. Int. Health.

[17] Rakholiya K.D., Kaneria M.J., Chanda S.V. (2013). Medicinal Plants as Alternative Sources of Therapeutics against Multidrug-Resistant Pathogenic Microorganisms Based on Their Antimicrobial Potential and Synergistic Properties. Fighting Multidrug Resistance with Herbal Extracts, Essential Oils and Their Components.

[18] Weerakkody N.S., Caffin N., Turner M.S., Dykes G.A. (2010). In Vitro Antimicrobial Activity of Less-Utilized Spice and Herb Extracts against Selected Food-Borne Bacteria. Food Control.

[19] Abdelmontaleb H.S., Abdelmeged D.A., Hamdy S.M., Hammam M.G., Ebid W.M.A. (2025). Exploring the Potential of Using Pomegranate and Mango Peel Powders as Natural Food Additives Targeting Safety of White Soft Cheese. Int. J. Food Microbiol..

[20] Chan C.-L., Gan R.-Y., Shah N.P., Corke H. (2018). Polyphenols from Selected Dietary Spices and Medicinal Herbs Differentially Affect Common Food-Borne Pathogenic Bacteria and Lactic Acid Bacteria. Food Control.

[21] Luo P., Feng X., Liu S., Jiang Y. (2024). Traditional Uses, Phytochemistry, Pharmacology and Toxicology of *Ruta graveolens* L.: A Critical Review and Future Perspectives. Drug Des. Dev. Ther..

[22] Almasri R.S., Bedir A.S., Al Raish S.M. (2025). Comprehensive Ethnopharmacological Analysis of Medicinal Plants in the UAE: *Lawsonia inermis*, *Nigella sativa*, *Ziziphus spina-christi*, *Allium cepa*, *Allium sativum*, *Cymbopogon schoenanthus*, *Matricaria aurea*, *Phoenix dactylifera*, *Portulaca oleracea*, *Reichardia tingitana*, *Salvadora persica*, *Solanum lycopersicum*, *Trigonella foenum-graecum*, *Withania somnifera*, and *Ziziphus lotus*. Nutrients.

[23] Elnady R.E., Abdon M.S., Shaheen H.R., Eladawy R.M., Azar Y.O., Al Raish S.M. (2025). The Future of Alopecia Treatment: Plant Extracts, Nanocarriers, and 3D Bioprinting in Focus. Pharmaceutics.

[24] Al Raish S.M., Almasri R.S., Bedir A.S. (2025). Ancient Remedies, Modern Medicine: A Review of Antidiabetic, Cardioprotective, and Antimicrobial Activities of Date Palm (*Phoenix dactylifera*), Tomato (*Solanum lycopersicum*), Fenugreek (*Trigonella foenum-graecum*), and Ashwagandha (*Withania somnifera*). Biology.

[25] Shokr M.M., Eladawy R.M., Azar Y.O., Al Raish S.M. (2025). Probiotics and the Gut–Brain Axis: Emerging Therapeutic Strategies for Epilepsy and Depression Comorbidity. Foods.

[26] Bedir A.S., Almasri R.S., Azar Y.O., Elnady R.E., Al Raish S.M. (2025). Exploring the Therapeutic Potential of Allium Cepa and Allium Sativum Extracts: Current Strategies, Emerging Applications, and Sustainability Utilization. Biology.

[27] Abdul Aziz A.H., Rizkiyah D.N., Qomariyah L., Irianto I., Che Yunus M.A., Putra N.R. (2023). Unlocking the Full Potential of Clove (Syzygium Aromaticum) Spice: An Overview of Extraction Techniques, Bioactivity, and Future Opportunities in the Food and Beverage Industry. Processes.

[28] Al Raish S.M., Almasri R.S., Bedir A.S., Elkahwagy A.A. (2025). Phytochemical Composition, Bioactive Compounds, and Antidiabetic Potential of Four Medicinal Plants Native to the UAE: *Capparis spinosa*, *Citrullus colocynthis*, *Morus alba*, and *Rhazya stricta*. Biology.

[29] Bedir A.S., Almasri R.S., Al Raish S.M. (2025). Therapeutic Efficacy of Nigella Sativa and Ziziphus Lotus: Sustainable Strategies for Diabetes, Antimicrobial Resistance, and Health Treatment. Front. Nutr..

[30] Gengatharan A., Rahim M.H.A. (2023). The Application of Clove Extracts as a Potential Functional Component in Active Food Packaging Materials and Model Food Systems: A Mini-Review. Appl. Food Res..

[31] Idowu S., Adekoya A.E., Igiehon O.O., Idowu A.T. (2021). Clove (*Syzygium aromaticum*) Spices: A Review on Their Bioactivities, Current Use, and Potential Application in Dairy Products. Food Meas..

[32] El-Maati M.F.A., Mahgoub S.A., Labib S.M., Al-Gaby A.M.A., Ramadan M.F. (2016). Phenolic Extracts of Clove (*Syzygium aromaticum*) with Novel Antioxidant and Antibacterial Activities. Eur. J. Integr. Med..

[33] Ju J., Xu X., Xie Y., Guo Y., Cheng Y., Qian H., Yao W. (2018). Inhibitory Effects of Cinnamon and Clove Essential Oils on Mold Growth on Baked Foods. Food Chem..

[34] Hateet R., Hachim A., Shawi H. (2016). Biological Activity of Eugenol Acetate as Antibacterial and Antioxidant Agent, Isolation from *Myrtus communis* L. Essential Oil. Int. J. Bioeng. Biotechnol..

[35] Pichette A., Larouche P., Lebrun M., Legault J. (2006). Composition and Antibacterial Activity of *Abies balsamea* Essential Oil. Phytother. Res..

[36] Melgarejo-Sánchez P., Núñez-Gómez D., Martínez-Nicolás J.J., Hernández F., Legua P., Melgarejo P. (2021). Pomegranate Variety and Pomegranate Plant Part, Relevance from Bioactive Point of View: A Review. Bioresour. Bioprocess..

[37] Moradi M.-T., Karimi A., Shahrani M., Hashemi L., Ghaffari-Goosheh M.-S. (2019). Anti-Influenza Virus Activity and Phenolic Content of Pomegranate (*Punica granatum* L.) Peel Extract and Fractions. Avicenna J. Med. Biotechnol..

[38] Nazeam J.A., AL-Shareef W.A., Helmy M.W., El-Haddad A.E. (2020). Bioassay-Guided Isolation of Potential Bioactive Constituents from Pomegranate Agrifood by-Product. Food Chem..

[39] Sun S., Huang S., Shi Y., Shao Y., Qiu J., Sedjoah R.-C.A.-A., Yan Z., Ding L., Zou D., Xin Z. (2021). Extraction, Isolation, Characterization and Antimicrobial Activities of Non-Extractable Polyphenols from Pomegranate Peel. Food Chem..

[40] Pacheco-Quito E.-M., Avila-Cunalata D., Cuenca-León K. (2025). Cariostatic Agents: From Silver Diamine Fluoride to Emerging Bioactive Compounds. Clin. Cosmet. Investig. Dent..

[41] Gong S., Fei P., Sun Q., Guo L., Jiang L., Duo K., Bi X., Yun X. (2021). Action Mode of Cranberry Anthocyanin on Physiological and Morphological Properties of *Staphylococcus aureus* and Its Application in Cooked Meat. Food Microbiol..

[42] Côté J., Caillet S., Doyon G., Dussault D., Sylvain J.-F., Lacroix M. (2011). Antimicrobial Effect of Cranberry Juice and Extracts. Food Control.

[43] Samarasinghe S., Reid R., Al-Bayati M. (2019). The Anti-Virulence Effect of Cranberry Active Compound Proanthocyanins (PACs) on Expression of Genes in the Third-Generation Cephalosporin-Resistant *Escherichia coli* CTX-M-15 Associated with Urinary Tract Infection. Antimicrob. Resist. Infect. Control.

[44] Das G., Gonçalves S., Basilio Heredia J., Romano A., Jiménez-Ortega L.A., Gutiérrez-Grijalva E.P., Shin H.S., Patra J.K. (2022). Cardiovascular Protective Effect of Cinnamon and Its Major Bioactive Constituents: An Update. J. Funct. Foods.

[45] Miron T., Rabinkov A., Mirelman D., Wilchek M., Weiner L. (2000). The Mode of Action of Allicin: Its Ready Permeability through Phospholipid Membranes May Contribute to Its Biological Activity. Biochim. Biophys. Acta (BBA)-Biomembr..

[46] Liaqat A., Zahoor T., Atif Randhawa M., Shahid M. (2019). Characterization and Antimicrobial Potential of Bioactive Components of Sonicated Extract from Garlic (*Allium sativum*) against Foodborne Pathogens. J. Food Process Preserv..

[47] Bhatwalkar S.B., Mondal R., Krishna S.B.N., Adam J.K., Govender P., Anupam R. (2021). Antibacterial Properties of Organosulfur Compounds of Garlic (*Allium sativum*). Front. Microbiol..

[48] Jain M., Patil N., Mohammed A., Hamzah Z. (2025). Valorization of Garlic (*Allium sativum* L.) Byproducts: Bioactive Compounds, Biological Properties, and Applications. J. Food Sci..

[49] Cao W., Li L., Wang J., Guo W., Chen W., Pan L., Li D. (2025). Effects of Black Garlic Polyphenols on the Physicochemical Characteristics, Antioxidant Activity, and Sensory Evaluation of Yogurt. Gels.

[50] Ramachandran E., Dhanapal S., Gopika U.G., Ariyamuthu R. (2025). From Spice to Mind: How Cinnamon Supports Women’s Mental Health. J. Ayurveda Holist. Med..

[51] Almatroodi S.A., Alsahli M.A., Almatroudi A., Anwar S., Verma A.K., Dev K., Rahmani A.H. (2020). Cinnamon and Its Active Compounds: A Potential Candidate in Disease and Tumour Management through Modulating Various Genes Activity. Gene Rep..

[52] Khan K., Al-Khalaifah H., Ahmad N., Khan M.T., Alonaizan R., Khan R.U., Naz S., Abudabos A., Alhidary I.A. (2025). Dietary Supplementation of Cinnamon and Turmeric Powder Enhances Growth Performance, Nutrient Digestibility, Immune Response, and Renal Function in Broiler Chickens. Poult. Sci..

[53] Aguilar-Zarate P., Wong-Paz J.E., Buenrostro-Figueroa J.J., Ascacio J.A., Contreras-Esquivel J.C., Aguilar C.N. (2018). Ellagitannins: Bioavailability, Purification and Biotechnological Degradation. Mini Rev. Med. Chem..

[54] Jeyakumar G.E., Lawrence R. (2021). Mechanisms of Bactericidal Action of Eugenol against *Escherichia coli*. J. Herbal. Med..

[55] Ramadan M.F. (2022). Clove (Syzygium aromaticum): Chemistry, Functionality and Applications.

[56] Howell A.B., D’Souza D.H. (2013). The Pomegranate: Effects on Bacteria and Viruses That Influence Human Health. Evid.-Based Complement. Altern. Med..

[57] Pérez C., Anesini C. (1994). In Vitro Antibacterial Activity of Argentine Folk Medicinal Plants against *Salmonella typhi*. J. Ethnopharmacol..

[58] Kon K.V., Rai M.K. (2012). Plant Essential Oils and Their Constituents in Coping with Multidrug-Resistant Bacteria. Expert. Rev. Anti-Infect. Ther..

[59] Salim A., Deiana P., Fancello F., Molinu M.G., Santona M., Zara S. (2023). Antimicrobial and Antibiofilm Activities of Pomegranate Peel Phenolic Compounds: Varietal Screening through a Multivariate Approach. J. Bioresour. Bioprod..

[60] Wu V.C.-H., Qiu X., Bushway A., Harper L. (2008). Antibacterial Effects of American Cranberry (*Vaccinium macrocarpon*) Concentrate on Foodborne Pathogens. LWT-Food Sci. Technol..

[61] Tamkutė L., Vaicekauskaitė R., Gil B.M., Rovira Carballido J., Venskutonis P.R. (2021). Black Chokeberry (*Aronia melanocarpa* L.) Pomace Extracts Inhibit Food Pathogenic and Spoilage Bacteria and Increase the Microbiological Safety of Pork Products. J. Food Process. Preserv..

[62] Boira C., Jolibois J., Durduret A., Tiguemounine J., Szewezyk C., De Tollenaere M., Scandolera A., Reynaud R. (2025). Cranberry Oil: A Potent Natural Intimate Care Ingredient Displaying Antioxidant and Anti-Inflammatory Effects and Promoting Beneficial Vaginal Lactobacillus. Int. J. Mol. Sci..

[63] Alqasoumi S. (2012). Anti-Secretagogue and Antiulcer Effects of Cinnamon *Cinnamomum zeylanicum* in Rats. J. Pharmacog. Phytother..

[64] Rashid A., Qayum A., Bacha S.A.S., Liang Q., Liu Y., Kang L., Chi Z., Chi R., Han X., Ekumah J.-N. (2024). Preparation and Functional Characterization of Pullulan-Sodium Alginate Composite Film Enhanced with Ultrasound-Assisted Clove Essential Oil Nanoemulsions for Effective Preservation of Cherries and Mushrooms. Food Chem..

[65] Mia M.S., Ara R., Rahman O., Shaha L.C., Galib R.M., Alam M. (2025). Valorization of Fruit Peel Extracts as Natural Preservatives: Characterization and Efficacy in Preserving Chicken Meatballs. Appl. Food Res..

[66] Jany J.F., Nupur A.H., Akash S.I., Karmoker P., Mazumder M.A.R., Alim M.A. (2024). Fortification of Functional Yogurt by the Phytochemicals Extracted from Pomegranate Peel. Appl. Food Res..

[67] Sallam K.I., Raslan M.T., Sabala R.F., Abd-Elghany S.M., Mahros M.A., Elshebrawy H.A. (2024). Antimicrobial Effect of Garlic against Foodborne Pathogens in Ground Mutton. Food Microbiol..

[68] Ahmed N., El-Fateh M., Amer M.S., El-Shafei R.A., Bilal M., Diarra M.S., Zhao X. (2023). Antioxidative and Cytoprotective Efficacy of Ethanolic Extracted Cranberry Pomace against Salmonella Enteritidis Infection in Chicken Liver Cells. Antioxidants.

[69] Gülçin İ., Elmastaş M., Aboul-Enein H.Y. (2012). Antioxidant Activity of Clove Oil—A Powerful Antioxidant Source. Arab. J. Chem..

[70] Côté J., Caillet S., Doyon G., Sylvain J.-F., Lacroix M. (2010). Bioactive Compounds in Cranberries and Their Biological Properties. Crit. Rev. Food Sci. Nutr..

[71] Błaszczyk N., Rosiak A., Kałużna-Czaplińska J. (2021). The Potential Role of Cinnamon in Human Health. Forests.

[72] Sharokhi Rezaei S., Nouri L., Mazaheri M., Adlnasab L. (2025). Mycotoxin Control Using Natural Solutions: Assessing Cinnamon Extract’s Impact on Aflatoxin Production. Iran. J. Chem. Chem. Eng..

[73] Dong J., Fan Z., Luo T., Deng C., Zheng X., Cai C., Ou R., Liu Z., Liu T., Wang Q. (2025). Dual-Mode Synergistic Antibacterial Films Based on Photothermal Effect and Controlled Release of Cinnamon Essential Oil Microcapsules for Food Packaging. Chem. Eng. J..

[74] Jayathilake A.L., Jayasinghe M.A., Walpita J. (2022). Development of Ginger, Turmeric Oleoresins and Pomegranate Peel Extracts Incorporated Pasteurized Milk with Pharmacologically Important Active Compounds. Appl. Food Res..

[75] Iqbal S., Haleem S., Akhtar M., Zia-ul-Haq M., Akbar J. (2008). Efficiency of Pomegranate Peel Extracts in Stabilization of Sunflower Oil under Accelerated Conditions. Food Res. Int..

[76] Česonienė L., Labokas J., Jasutienė I., Šarkinas A., Kaškonienė V., Kaškonas P., Kazernavičiūtė R., Pažereckaitė A., Daubaras R. (2021). Bioactive Compounds, Antioxidant, and Antibacterial Properties of Lonicera Caerulea Berries: Evaluation of 11 Cultivars. Plants.

[77] Palombo E.A. (2006). Phytochemicals from Traditional Medicinal Plants Used in the Treatment of Diarrhoea: Modes of Action and Effects on Intestinal Function. Phytother. Res..

[78] Malviya S., Arvind, Jha A., Hettiarachchy N. (2014). Antioxidant and Antibacterial Potential of Pomegranate Peel Extracts. J. Food Sci. Technol..

[79] Park S.-Y., Kim Y.D., Kim M.S., Kim K.-T., Kim J.Y. (2023). Cinnamon (*Cinnamomum cassia*) Water Extract Improves Diarrhea Symptoms by Changing the Gut Environment: A Randomized Controlled Trial. Food Funct..

[80] Qnais E.Y., Elokda A.S., Abu Ghalyun Y.Y., Abdulla F.A. (2007). Antidiarrheal Activity of the Aqueous Extract of *Punica granatum* (Pomegranate) Peels. Pharm. Biol..

[81] Imani G., Khalilian A., Dastan D., Imani B., Mehrpooya M. (2019). Effects of Cinnamon Extract on Complications of Treatment and Eradication of Helicobacter Pylori in Infected People. J. Herbmed. Pharmacol..

[82] Yeomans N.D., Naesdal J. (2008). Systematic Review: Ulcer Definition in NSAID Ulcer Prevention Trials. Aliment. Pharmacol. Ther..

[83] Issac A., Gopakumar G., Kuttan R., Maliakel B., Krishnakumar I.M. (2015). Safety and Anti-Ulcerogenic Activity of a Novel Polyphenol-Rich Extract of Clove Buds (*Syzygium aromaticum* L). Food Funct..

[84] Abdel-Kawi S.H., Hashem K.S., Saad M.K., Fekry G., Abdel-Hameed E.M.M. (2022). The Ameliorative Effects of Cinnamon Oil against Ethanol-Induced Gastric Ulcer in Rats by Regulating Oxidative Stress and Promoting Angiogenesis. J. Mol. Histol..

[85] Alqirnawdi M.A.A., Khotimah H., Santosa S., Mintaroem K. (2020). The Effect of Cinnamon to the Expression of SOD-1 and TNF-α in Indomethacin-Induced Gastric Ulcer Rat. AIP Conf. Proc..

[86] Khan M.K., Hassan S., Imran M., Ahmad M.H., Ramadan M.F. (2022). Chapter 22—Extraction of Bioactive Compounds from Clove (*Syzygium aromaticum*). Clove (Syzygium aromaticum): Chemistry, Functionality and Applications.

[87] Haro-González J.N., Castillo-Herrera G.A., Martínez-Velázquez M., Espinosa-Andrews H. (2021). Clove Essential Oil (*Syzygium aromaticum* L. Myrtaceae): Extraction, Chemical Composition, Food Applications, and Essential Bioactivity for Human Health. Molecules.

[88] Severo C., Anjos I., Souza V.G.L., Canejo J.P., Bronze M.R., Fernando A.L., Coelhoso I., Bettencourt A.F., Ribeiro I.A.C. (2021). Development of Cranberry Extract Films for the Enhancement of Food Packaging Antimicrobial Properties. Food Packag. Shelf Life.

[89] Sridhar A., Ponnuchamy M., Kumar P.S., Kapoor A. (2021). Food Preservation Techniques and Nanotechnology for Increased Shelf Life of Fruits, Vegetables, Beverages and Spices: A Review. Environ. Chem. Lett..

[90] Kumar S., Singh N., Devi L.S., Kumar S., Kamle M., Kumar P., Mukherjee A. (2022). Neem Oil and Its Nanoemulsion in Sustainable Food Preservation and Packaging: Current Status and Future Prospects. J. Agric. Food Res..

[91] Khalili M., Najafi A., Razavi R. (2025). Preservative Activity of Free and Nano-Encapsulated Pomegranate Peel Extract Obtained Using Cold Plasma and Ultrasound-Assisted Method in Increasing the Shelf Life of Thigh Mutton Mince. Appl. Food Res..

[92] Dong Y., Zhang J., Xie A., Yue X., Li M., Zhou Q. (2024). Garlic Peel Extract as an Antioxidant Inhibits Triple-Negative Breast Tumor Growth and Angiogenesis by Inhibiting Cyclooxygenase-2 Expression. Food Sci. Nutr..

